# Thyroid hormones act as modulators of inflammation through their nuclear receptors

**DOI:** 10.3389/fendo.2022.937099

**Published:** 2022-08-08

**Authors:** Marina Lasa, Constanza Contreras-Jurado

**Affiliations:** ^1^ Departamento de Bioquímica-Instituto de Investigaciones Biomédicas “Alberto Sols”, Universidad Autónoma de Madrid-Consejo Superior de Investigaciones Científicas, Madrid, Spain; ^2^ Departamento de Bioquímica, Facultad de Medicina, Universidad Alfonso X El Sabio, Madrid, Spain; ^3^ Departamento de Fisiopatología Endocrina y del Sistema Nervioso, Instituto de Investigaciones Biomédicas “Alberto Sols”, Consejo Superior de Investigaciones Científicas and Universidad Autónoma de Madrid, Madrid, Spain; ^4^ Centro de Investigación Biomédica en Red de Cáncer (CIBERONC), Instituto de Salud Carlos III (ISCIII), Madrid, Spain

**Keywords:** thyroid hormones, thyroid receptors, chronic inflammation, sepsis, cancer, signaling pathway

## Abstract

Reciprocal crosstalk between endocrine and immune systems has been well-documented both in physiological and pathological conditions, although the connection between the immune system and thyroid hormones (THs) remains largely unclear. Inflammation and infection are two important processes modulated by the immune system, which have profound effects on both central and peripheral THs metabolism. Conversely, optimal levels of THs are necessary for the maintenance of immune function and response. Although some effects of THs are mediated by their binding to cell membrane integrin receptors, triggering a non-genomic response, most of the actions of these hormones involve their binding to specific nuclear thyroid receptors (TRs), which generate a genomic response by modulating the activity of a great variety of transcription factors. In this special review on THs role in health and disease, we highlight the relevance of these hormones in the molecular mechanisms linked to inflammation upon their binding to specific nuclear receptors. In particular, we focus on THs effects on different signaling pathways involved in the inflammation associated with various infectious and/or pathological processes, emphasizing those mediated by NF-kB, p38MAPK and JAK/STAT. The findings showed in this review suggest new opportunities to improve current therapeutic strategies for the treatment of inflammation associated with several infections and/or diseases, such as cancer, sepsis or Covid-19 infection.

## Introduction

### Thyroid hormones and its mechanism of action

Thyroid hormones, triiodothyronine (T3) and thyroxine (T4), are important regulatory molecules in the human body with an essential role in growth, development, metabolism, and immune system. In fact, both, deficiency and excess of THs are associated with severe disorders affecting different organs (1). The principal hormone secreted by the human thyroid gland and released into the bloodstream is T4, which is converted by deiodination in peripheral tissues into the active hormone T3. Local THs concentrations are further adjusted by different deiodinases (DIOs). Three different DIOs are known, DIO1 and DIO2 catalyze the conversion of T4 into T3, being DIO2 the most efficient. Inactivation of THs is mediated by DIO3, that converts T4 and T3 into reverse-T3 (rT3) and 3,3’-T2, respectively (2). Metabolism and action of THs take place intracellularly by TH transporter proteins like monocarboxylate transporter (MCT)8, MCT10, and organic anion-transporting polypeptide 1C1 (3). Although THs can act by binding to different molecules located on plasma membrane, like integrin αvβ3 (4), most actions of the THs are mediated intracellularly by binding to **thyroid nuclear receptors** (TRs). T3 shows higher affinity than T4 for TRs, whereas T4 is more potent than T3 in binding integrin αvβ3. TRs act as ligand-dependent transcription factors by directly activating THs response elements (TREs) on gene promoters (canonical signaling). TRs may additionally act *via* noncanonical signaling, activating molecules such as phosphoinositide 3-phosphate kinase (PI3K), protein kinase B (AKT), and mitogen-activated protein kinases (MAPKs) (5). TRs are encoded by two different genes (α and β) located in human chromosomes 17 and 3, respectively. TRα1, TRβ1 and TRβ2 are the main hormone-binding isoforms (6). TR isoforms found in human, rat, and mouse tissues have highly homologous amino acid sequences and are expressed at different relative levels in diverse tissues (7). TRs share a common structure with other members of the nuclear receptor family (receptors for steroid hormones, retinoids, vitamin D, and different “orphan” receptors) (8), and they can bind to TREs as homodimers or preferentially as heterodimers with retinoid X receptor (RXR). As a consequence of all these molecular mechanisms, THs, *via* their binding to TRs, can regulate the expression of a wide number of target genes that play crucial roles in the brain, cardiovascular, skeletal muscle, hepatic, renal, and intestinal systems (9). Also, it has been highlighted bidirectional crosstalk between THs and the immune system (10). In recent years, there is growing evidence for a direct influence of THs on inflammation and diverse research groups have shown that THs regulate the transcription of many genes involved in different inflammatory-related pathways, including xanthine oxidase expression through TLR4/NF-kB pathway (11), IL-1β expression in macrophages (12), IL-6 signaling during endotoxemia (13), modulation of IL-17 in dendritic cells (14), activation of M1 cells through TRβ1 receptor (15), activation of S100A8/MyD88/NF-kB signaling pathway (16), induction of the phosphatase DUSP1 (17, 18), or inhibition of both NF-kB and p38MAPK signaling pathways in pituitary tumor cells (17, 18). For this reason, in this special review on THs role in health and disease, we focus on the relevance of these hormones in the molecular mechanisms linked to inflammation upon their binding to specific nuclear receptors. In particular, we highlight THs effects on different signaling pathways involved in the inflammation associated with various infectious and/or pathological processes, emphasizing those mediated by NF-kB, p38MAPK and/or JAK/STAT cascades.

### Molecular mechanisms involved in inflammation-related diseases

Inflammation is a physiological process generated by cells of innate and adaptative immunity in order to rebuild an injured tissue in response to damage triggered by a wide variety of chemical agents and conditions, such as infection (19). Immediately after the aggression, a response of short duration appears, dilutes and destroys the pathogen and, at the same time, initiates healing of injured tissue by regeneration to rid the body of the cause of the aggression. This type of reaction is known as **acute inflammatory response** and is generally managed by macrophages and mast cells, which lead to the production of a variety of inflammatory mediators, including chemokines and cytokines such as tumor necrosis factor-α (TNFα) and transforming growth factor-α (TGFα), vasoactive amines, and eicosanoids, among others (20). One of the main effects of these mediators is to activate the endothelium and to recruit neutrophils at the site of infection or injury, where they become active and release the toxic content of their granules, including reactive oxygen species, which induce the elimination of the infectious agent. This step is usually followed by a repair phase that involves the recruitment of monocytes, which remove dead cells and promote tissue remodeling by inducing the migration of vascular endothelium-specific molecules to sites of injury facilitated by extracellular proteases, such as matrix metalloproteinases (MMPs) (20). Hence, this process is essential not only in host defense against pathogens, but also in tissue regeneration, repair and homeostasis (19).

Several evidences have demonstrated that different signaling pathways are central mediators of the physiological inflammatory response. One of these signaling cascades is driven by the family of transcription factors called **NF-kB** (Nuclear Factor of the k-chain in B-cells). This protein family consist of five members, which form homo- or heterodimers in order to bind to enhancer elements in the promoter regions of target genes. Two members (p100 and p105) can be proteolytically processed and the resulting proteins (p52 and p50) need to dimerize with one of the other three family members, RelA (p65), RelB, or c-Rel, to function as transcription factors (21). One of the most common NF-kB dimers is formed by p65 bound to p50 members and remains inactive under basal conditions by association with a member of the IkB family, which mostly prevents translocation of NF-kB dimers into the nucleus. NF-kB activity can be triggered by the classical pathway in response to inflammatory cytokines such as TNFα or IL-1β, or to bacterial cell wall components like lipopolysaccharides (LPS) (21, 22); alternatively, the non-classical or non-canonical activation of NF-kB is achieved by CD40 ligand or lymphotoxin b; finally, NF-kB can also be activated under stress conditions such as UV-irradiation by the atypical signaling pathway (23). Although these pathways have been considered to work independently, it has been recently shown that stimulation of the non-canonical pathway can also activate components of the classical pathway, so the transcriptional responses can be qualitatively very similar (24). In general, ligands of NF-kB-activating pathways usually trigger conformational changes in specific receptors, which generate recruitment platforms of intracellular adaptor proteins, that converge in the activation of the IkB kinases, IKKα or IKKβ. Then, these proteins phosphorylate the inhibitory molecules of the IkB family or the inhibitory domains of p100 and p105, triggering the poly-ubiquitination and the subsequent proteasomal degradation of inhibitors. This leads to the translocation of NF-kB dimers into the nucleus, where they can regulate the transcription of target genes involved in different steps of the inflammatory response (25). In fact, this pathway can modulate the transcription of several pro-inflammatory cytokines and chemokines, adhesion molecules, anti-apoptotic genes, and enzymes such as cyclooxygenases (Cox) that catalyze prostaglandin formation. There is an additional mechanism of control of NF-kB activation based on the regulation of its transcriptional activity by post-translational modifications of some of the proteins involved in this signaling cascade. Thus, to obtain a maximum response, NF-kB must undergo phosphorylations and/or acetylations, which determine the intensity and duration of the signal through the regulation of its localization and binding to co-activators or co-repressors in transcription complexes (23). This allows connections established between this signaling cascade and others, such as the p38MAPK and JAK/STAT pathways, which can cooperate to maintain the inflammatory environment (25). Thus, the biological responses following NF-kB activation are highly dependent on the combination of different issues, such as the cell type, the regulated target genes and the epigenetic mechanisms which control their accessibility, as well as the possible crosstalk with other signaling cascades.

Another signaling pathway that is important for controlling the physiological inflammatory response is the **p38MAPK signaling cascade**. There are different p38MAPK isoforms, whose expression is either ubiquitous (α and β) or tissue-specific (γ and δ) (26). In general, most p38MAPK are activated through the canonical pathway, which starts with the induction of a group of kinases known as MAP3Ks in response to various stimuli, including cytokines, ligands of G protein-coupled receptors such as hormones, metabolites, and neurotransmitters, as well as stress signals. Once activated, MAP3Ks phosphorylate the MAP2Ks MKK3 and MKK6, which specifically catalyze the phosphorylation of threonine and tyrosine residues in the activation loop of p38MAPK (26). Alternatively, p38MAPKα can also be activated by the non-canonical pathway, which has been widely studied in different inflammation models and involves p38MAPK autophosphorylation upon its binding to TGFβ-activated kinase 1-binding protein 1 (27, 28). In any case, once p38MAPK is activated, many substrates are phosphorylated, either directly or through different downstream kinases. The complexity of the p38MAPK pathway increases with the identification of additional regulatory mechanisms, which involve either the modulation of enzymes responsible for post-translational modifications (29), the inactivation of the p38MAPK isoforms by dual-specificity phosphatases of the DUSP/MKP family (30) or the crosstalk with other signaling pathways. In this sense, we have demonstrated a connection with the NF-kB pathway in diverse cellular contexts (18, 31, 32). It is important to note that p38MAPK signaling cascade plays an important role in the inflammatory response, not only because it can be activated by pro-inflammatory cytokines (33), but also because it can regulate the production of different inflammatory mediators. In general, this regulation occurs through modulation of pro-inflammatory transcription factors, such as NF-kB (25, 34), or by regulating the stability of the mRNAs of different inflammation-related molecules (34–36).

The third main signaling cascade that plays a crucial role in the physiological inflammatory response is the **JAK/STAT signaling system**. This cascade is made up of different types of proteins, which are divided into three main groups according to their structure and function (37): (i) cytokine receptors, with a ligand-binding extracellular domain and an intracellular domain in their structures, which, unlike other membrane receptors, do not have catalytic activity; (ii) JAK (Janus Kinases) proteins, with two tyrosine kinase domains, one of them with catalytic activity; and (iii) STAT (signal transducer and activator of transcription) proteins, a family of transcription factors that have in their structure different domains responsible for their phosphorylation, dimer-dimer interaction and activation. In addition, both JAK and STAT proteins have also SH2 domains, through which they can interact with other proteins in the same or other signaling pathways (38). Regarding the activation of this pathway, in general, cytokine receptors are activated upon binding of various cytokines or growth factors to their extracellular domains, which cause a conformational change that induce their dimerization. Receptor dimers are then capable of recruiting one or more members of the JAK kinase protein family. As a result of this, JAK proteins become activated and promote cross-phosphorylation of the receptor to which they are bound, creating docking sites for different proteins, including the STATs, a family of transcription factors that reside in the cytoplasm until activated (39). STATs proteins are then phosphorylated, which allow their dimerization and subsequent translocation to the nucleus, where they regulate the transcription of genes encoding for proteins involved in inflammatory processes (38). Although the mechanism of JAK/STAT signaling pathway is relatively simple, the biological consequences of its activation are complicated by interactions with other signaling cascades. Hence, phosphorylation of cytokine receptors by JAK proteins also allows their interaction with SH2 domain-proteins that belong to other signaling systems (40). Alternatively, STAT proteins can physically interact with other proteins, such as components of the NF-kB signaling cascade (41), or can be transcriptionally activated by other proteins, such as p38MAPK (42).

Although the acute inflammatory response is usually transient and beneficial against infection and tissue injury, sometimes inflammation is maintained over time, and become detrimental due to the activation of simultaneous processes that include destruction and healing of damaged tissue. This kind of inflammation is called **chronic inflammation** and is typical of diseases such as some types of cancer (43), and other disorders including rheumatoid arthritis, atherosclerosis or Crohn’s disease (44). In fact, it is estimated that about 15% of human cancers are associated with chronic infection and inflammation (45). Chronic inflammation can be caused not only by the classical inflammatory activators, such as infection and injury, but also by the homeostatic imbalance of several physiological systems (19). This type of inflammation is characterized by the proliferation of blood vessels, the progressive change of the cell type at the inflammation site, the proliferation of fibroblasts, the increase in connective tissue with the appearance of fibrosis and, finally, tissue necrosis and destruction. At the molecular level, most of the inflammatory-related diseases exhibit alterations in the functioning of the signaling pathways mentioned above. For instance, **NF-kB signaling pathway** has been found to be basally hyperactivated in many types of inflammation-linked tumors (25); in this sense, our group and others have shown that NF-kB hyperactivation in prostate, pituitary, kidney or liver tumors can be explained by different molecular mechanisms, which involve a basal activation of IKKs, a nuclear localization of NF-kB dimers in the absence of stimulus, and/or a basal activation of genes dependent on this transcription factor, such as Cox-2 (17, 18, 46–49). In addition, this signaling cascade is also altered in the pathogenesis of other inflammatory contexts, such as the excitotoxicity associated to neurodegeneration (32) or the inflammation linked to a number of diseases, including rheumatoid arthritis, multiple sclerosis, chronic obstructive pulmonary disease or asthma, among others (50). On the other hand, the **p38MAPK signaling pathway** is also activated at baseline in various tumors and inflammatory contexts mainly due to impairment of the inhibitory feedback mechanisms that usually reverse activation. For example, bacterial infection triggers the activation of p38MAPKα either through the canonical pathway or the inactivation of certain phosphatases (26), while severe acute respiratory syndrome coronavirus 2 (SARS-CoV-2) infection induces late activation of this p38MAPK isoform, suggesting its implication in advanced stages of viral infection (51). Moreover, activation of p38MAPK pathway has been often correlated with cardiac pathologies, in which inflammation plays an important role (52). Regarding cancer, p38MAPKα signaling has been shown to support tumor growth by facilitating the inflammation regulated by macrophages and dendritic cells in colon cancer (53, 54). Furthermore, non-canonical p38MAPKα activation in T cells promotes an inflammatory state that facilitates pancreatic ductal carcinoma development (55), while breast cancer cells also rely on p38MAPKα to produce cytokines and chemokines that recruit pro-tumorigenic myeloid cells to the tumor niche (56). Finally, the **JAK/STAT signaling pathway** has also been persistently activated in several inflammatory pathologies. For instance, it has been demonstrated that the blockade of JAK2/STAT3 signaling pathway notably inhibits the protein levels of high mobility group box 1, an important mediator in the pathogenesis of many diseases, including arthritis, sepsis, cancer or autoimmunity diseases (57). Regarding cancer, different mutations have been identified in the genes encoding diverse JAK proteins in several types of tumors. These mutations generate constitutively active JAK proteins that, upon their binding to cytokine receptors, maintain in an active state the STAT proteins, promoting oncogenic transformation (41). Consistently, STAT3 is persistently activated and is required for cell transformation in melanoma, multiple myeloma, or in tumors of the breast, ovary, prostate, or colon (58). On the other hand, the hyperactivation of JAK/STAT signaling cascade has also been identified in other inflammation-related diseases. Interestingly, the crucial role of this cascade in rheumatoid arthritis has been recently established following the approval of the JAK3-selective small-molecule inhibitor, tofacitinib, for the medical therapy of this disease (59).

### Crosstalk between thyroid hormones and inflammation-linked diseases

Thyroid hormones regulate inflammasome and cancer cell growth in an opposite way, depending on their binding to integrin αvβ3 or TRs. Physiological levels of T3 in macrophages promote anti-inflammatory responses, and bactericidal and phagocytosis activity, through the binding to the TRs. In response to this merger, there is a downregulation of TLR4, NF-kB, NLRP3, pro-IL-1β, and pro-IL-18. But then, in a hypothyroidism condition, the high levels of T4 bind to plasma membrane integrin αvβ3 and activate the PI3K–AKT signaling cascade, which causes a robust production of ROS which triggers NLRP3 inflammasome and pro-inflammatory cytokines release (60). By binding to integrin αvβ3, thyroid hormones (mostly T4), induce activation of ERK1/2, PI3K, and STAT3 in different types of cancer cells (61) as has been shown in colorectal carcinoma (CRC) (62). We highlight the relevance of these hormones in the molecular mechanisms linked to inflammation upon their binding to specific nuclear receptors.

### Crosstalk between thyroid hormones and infection

It is well known that THs affect the innate and adaptive immune response during infection although the molecular mechanisms are not well elucidated. Clinical and preclinical studies have revealed that THs regulate the activity of neutrophils, macrophages, natural killer cells, dendritic cells (innate immune system) and B- and T-lymphocytes (adaptive immune system). In general, a hyperthyroid state leads to a more activated immune system whereas hypothyroidism leads to a less activated immune system (63).

When pathogen infections occur, the host immune system responds depending on the condition of the host and the magnitude of the attack. When the host’s protective innate immunity is deregulated and overactive, a reaction called **sepsis** occurs, in which pro-inflammatory signaling molecules are secreted and enter the bloodstream in large quantities, causing widespread, extensive and potentially fatal damage. If sepsis continues, hypoxia and organ damage develop into organ failure, which can lead to death. Most of the time, the infection that causes sepsis is bacterial (being bacterial peritonitis and pneumonia the two most common causes of sepsis), although it can also be triggered by fungal, viral and protozoal infections (64). The intracellular signaling system of sepsis is induced by binding of both pathogen-associated molecular patterns (PAMPs) or damage-associated molecular patterns (DAMPs) to complement, Toll-like receptors, nucleotide-binding oligomerization domain-like receptors, retinoic acid-inducible gene-like receptors, the mannose-binding lectin and scavenger receptors, among others. The activation of these signaling pathways leads to the recruitment of pro-inflammatory intermediates. This gives rise to the phosphorylation and activation of MAPKs, JAK/STATs and/or NF-kB. As a result of this induction, the expression of multiple early activation genes begins, including those encoding cytokines associated with inflammation like TNF, IL-1, IL-12, IL-18 and interferon (IFN). This event initiates a cascade of other inflammatory cytokines and chemokines including IL-6, IL-8, IFNγ, CC-chemokine ligand 2 (CCL2), CCL3 and CXC-chemokine ligand 10 (CXCL10), as well as the polarization and suppression of components of adaptive immunity. One of the hallmarks of sepsis is the complement activation which is initiated immediately upon exposure to PAMPs and DAMPs. Complement activation leads to the generation of complement peptides (C3a and C5a) during sepsis. C5a is the most active inflammatory peptide due to its potent action in attracting neutrophils, monocytes, and macrophages and its stimulation of the synthesis and release of pro-inflammatory cytokines and chemokines. As a consequence of that action, the inflammatory responses are amplified. Another hallmark of sepsis is immuno-suppression which occurs both early and late in the host sepsis response (65). Uncontrolled activation of both pro- and anti-inflammatory responses can lead to cell exhaustion, organ dysfunction, and death. The most lethal form of sepsis is called endotoxic shock, which is caused by LPS, the main component of the membrane of Gram-negative bacteria. During sepsis, thyroidal T4 and T3 synthesis are down-regulated by cytokines (66) and the severity of illness is reflected in the magnitude of the decrease in serum T3. In addition, the expression of DIOs responsible for THs metabolism during sepsis is also altered (67–69), and TRβ and RXR expression is reduced (70, 71).

It has been described a protective role of THs during inflammation by controlling the maturation and function of macrophages (15). As previously mentioned, macrophages play key roles in innate and adaptive immunity. There are two distinct states of polarized activation for macrophages, the classically (M1) and the alternatively (M2) activated macrophage phenotype. The M1 macrophages are activated by Toll-like receptor ligands, such as LPS and IFNγ, express pro-inflammatory cytokines, mediate the defense of the host from infection, and have roles in anti-tumor immunity. The M2 macrophages are stimulated by IL-4 or IL-13 and have anti-inflammatory, pro-tumoral functions and regulate wound healing. Macrophages, at different stages of growth, only express the receptor TRβ1 and the action of T3 through this receptor plays an important role in the regulation of growth/development and functional phenotype, as well as its influence on M1 or M2 activation in basal conditions and during systemic inflammation. In addition, Perrota et al. have demonstrated an *in vitro* negative role of T3, triggering the differentiation of bone marrow-derived monocytes into unpolarized macrophages, and the macrophages induced by T3 are M1 type (15). *In vivo* experiments revealed that T3 also significantly protects mice against endotoxemia induced by LPS. Thus, after LPS treatment, T3 levels decrease and, consequently, the recruitment of monocyte-derived cells, which are potentially damaging cells, is increased. However, when T3 is restored to normal levels in these animals, the number of resident cells is increased, and this effect is potentially beneficial (15). These results suggest an anti-inflammatory effect of T3 and demonstrates its protective role against the systemic inflammatory response of endotoxemia. A few years later, Furuya et al. found that ligand-bound TRα on macrophages plays a protective role in kidney inflammation through the inhibition of NF-kB pathway. They proved that T3 inhibits the nuclear localization of p65 in macrophages through a mechanism that enhances the stabilization of DUSP1 protein expression *via* MAPK, leading to down-regulation of NF-kB activity and expression of pro-inflammatory cytokines (12).

Interestingly, our group have described an important role of THs, through their binding to specific nuclear receptors, as potent regulators of immune homeostasis during sepsis, by directly repressing the response of the cells to inflammatory mediators. This action is carried out by the suppression of the signaling mediated by the cytokine IL-6 in macrophages and hepatocellular carcinoma cells (HCC), through STAT3 inhibition (13). This molecular mechanism proposes that the reduced responses to IL-6 should serve as a negative feedback mechanism for preventing deleterious effects of excessive hormone signaling during infections. *In vivo* experiments show that there are changes in p65 phosphorylation in the livers of knock-out TR mice, that suggest that the liganded receptors could also antagonize NF-kB activation (13), as previously described in other cellular systems (17, 18). In line with the data shown above, a recent meta-analysis study has been recently carried out to evaluate the association between thyroid disease and the outcome of COVID-19 patients (72). The regulation of the immune system plays an integral part in determining COVID-19 patients’ disease progressivity and it is well known that THs affect the immune response during infection. This study has evaluated hospital-based data from 21 studies published in scientific articles with a total of 31339 patients. Although the authors themselves acknowledge that the study has several limitations, the results of this analysis have revealed that thyroid disorder (abnormal thyroid and hypothyroidism, but not hyperthyroidism) is associated with increased composite poor COVID-19 outcome (higher risk ratio, disease severity, hospitalization, and intensive care unit admission). Other meta-analysis studies showing the same results (worse prognosis in patients with low T3 levels) have been published, proposing T3 as a tool for stratified management of patients with severe COVID-19 (73, 74).

### Crosstalk between thyroid hormones and cancer

The exact contribution of THs to cancer development and progression is unclear due to a large number of conflicting results available in the literature, depending on the cellular context or the transformation status (75). Thus, different reports have shown that the development of a wide variety of cancers, including thyroid, breast or prostate tumors is associated with high TH serum levels (76, 77). By contrast, subclinical hypothyroidism has also been identified as a predisposing risk factor for HCC (78), as well as certain thyroid, breast, bone or skin cancers (79). Of particular interest is the dual role that thyroid status plays in the development of T-cell lymphomas. Thus, on the one hand, hyperthyroidism favors tumor growth, while, on the other hand, hypothyroidism increases tumor spread (80–82). Many evidences have demonstrated that the role of THs as inducers of carcinogenesis and cancer progression is mainly mediated by non-genomic actions initiated at the cell membrane, through their interaction with the cell adhesion molecule, integrin αvβ3, that supports angiogenesis (83), and activates cellular proliferation *via* the induction of the ERK and/or PI3K signaling pathways (84–87). By contrast, a number of reports have supported the notion that restricted thyroid function favors cancer metastasis by mechanisms involving the binding of THs to their specific nuclear receptors, TRs. In fact, it has been shown that TRβ mainly mediates the inhibitory effect of THs on several processes that control tumors, such as proliferation, transformation, progression, invasion or metastasis (88–91). An interesting situation is that observed in different models of skin cancer. On the one hand, the intracellular regulation of TH levels by the inactivating deiodinase enzyme DIO3 seems to be critical for the tumorigenic potential of basal cell carcinomas (BCC). In addition, T3 attenuates BCC cell proliferation and tumorigenesis through its binding to TRβ (92). By contrast, in squamous cell carcinoma (SCC) cells, suppression of expression of the TH activating DIO2 deiodinase enzyme, causes enhanced cell growth, but also attenuates migration. Furthermore, in this model, T3 increases invasiveness and metastatic propensity through a mechanism involving its binding to TRα (93). Finally, the lack of both TRα and TRβ has also been shown to play a dual role in tumor development, because it restricts benign tumor formation at early stages of skin carcinogenesis but enhances malignant transformation at the later stages of the disease (94). This apparent controversy can be explained considering that, contrary to the well-accepted role of TRβ as a tumor suppressor, TRα has been shown to have oncogenic effects in diverse tumors (91, 95, 96).

The functional relationship between cancer and inflammation is supported by the fact that sustained cell proliferation in an environment rich in inflammatory cells, growth factors, and DNA-damage-promoting agents, can potentiate tumor initiation, promotion and/or progression (97). In this scenario, our group and others have shown that THs, upon their binding to specific nuclear receptors, are capable of regulating carcinogenesis and progression of a wide variety of tumors at different levels. Regarding **tumor initiation and promotion**, it has been shown that tumor cells can produce cytokines and chemokines that attract leukocytes, which in turn induce the expression of cytotoxic mediators, MMPs, cytokines, and soluble mediators of cell killing, contributing to the required accumulated mutations (43). In this sense, different mutations that either inactivate or reduce the expression of TRs have been identified in several tumors, including HCCs, renal cell carcinomas, and papillary thyroid carcinomas (98–100). In most cases, TRβ mutant variants found in tumors have a dominant-negative activity, impairing T3 binding or altering TRE recognition, and escape from regulatory mechanisms, promoting deregulated expression of target genes and cancer progression (101). In addition, elevated levels of cytokines such as IL-6 or TNFα observed in certain tumor contexts can also induce epigenetic alterations, affecting to DNA components and histones, and modulating the expression of oncogenes and tumor suppressors through the NF-kB, JAK/STAT or p38MAPK pathways (43). A number of reports have shown that TRs can affect these signaling cascades, thus regulating the initiation and promotion of different tumoral contexts. For example, we have demonstrated that T3 is able to induce apoptosis and impair the signaling mediated by the pro-inflammatory cytokine TNFα in pituitary tumor cells by a mechanism involving the inhibition of both NF-kB and p38MAPK signaling pathways (17, 18). In fact, this hormone decreases NF-kB-dependent transcription, IkBα phosphorylation, as well as nuclear translocation, phosphorylation, and transactivation of p65/NF-kB (17). Moreover, T3 also abolishes both TNFα-induced p38MAPK and NF-kB by a mechanism involving the induction of DUSP1 (18). The relevance of these results is supported by the fact that the pro-inflammatory cytokine TNFα has been shown to act as a tumor promoter in early events in tumors, regulating a cascade of cytokines, chemokines, adhesions, MMPs and pro-angiogenic activities (102). Other studies show that T3 inhibits HCC proliferation through the upregulation of the tumor suppressor p21 (103), which has been identified as a NF-kB target gene (104). In addition, T3 suppresses STAT5-mediated gene expression and inhibits mammary hyperplasia development (105). All these data indicate that T3 is able to inhibit processes implicated in the tumoral initiation and promotion by impairing different master regulatory pathways of inflammation.

Many reports have also shown a crucial role of TRs on **tumor progression and metastasis**. Inflammation influences cancer invasion, epithelial-mesenchymal transition (EMT), and cell migration on several levels. Thus, cytokines can directly affect the expression of EMT-inducing transcription factors (106). Alternatively, cytokines can mediate the recruitment of TGFβ-driven and -producing fibroblasts, known as tumor-associated macrophages, which support tumor invasion and immune escape in a number of tumors with the worst clinical prognosis (43). On the other hand, the proportion of cancer stem cells (CSCs), which are considered essential for tumor metastasis, increases in response to various stimuli, including prominent inflammatory signaling *via* transcription factors NF-kB and STAT3 (107, 108). Furthermore, inflammation also regulates the processes of intravasation and extravasation that are essential in the metastatic spread, *via* the expression of a set of adhesion molecules, integrins, inflammatory cytokines and growth factors like Vascular Endothelial Growth Factors (VEGF), among others (43). Different groups have demonstrated that TRs modulate progression and/or metastasis of different tumors through several mechanisms, mostly of which are connected to the inflammatory pathways activated in the tumoral environment (90). Among them, TRβ can inhibit tumor cell migration, invasion and metastasis in both cellular and animal models of HCC and breast cancer by antagonizing the actions of the TGFβ pathway (94, 109), which induces EMT in a p38MAPK-dependent manner in some tumors (110). Furthermore, TRβ inhibits the expression of pro-metastatic and pro-inflammatory genes such as Cox-2, MMP2, MMP9 and several chemokines (94). This effect is attenuated in the absence of the corepressor N-CoR (111), which has been shown to be dissociated from the chromatin upon its phosphorylation by IKKα (112). Consequently, the expression of both N-CoR and TRβ transcripts show a strong correlation and are significantly down-regulated in HCC and in the more aggressive breast tumors (113). More recently, it has been shown that TRβ is also able to limit the CSCs population in breast cancer by decreasing, not only their self-renewal capacity, but also the efficiency of mammosphere formation as well as the expression of pluripotency factors (114). Different reports have demonstrated that mammospheres are enriched in EMT markers such as the transcription factor Snail (115–117), which is constitutively upregulated in a wide variety of tumors (118), including prostate cancer (119), where NF-kB and p38MAPK signaling pathways play important roles (46). Despite this, TRβ increases Snail expression in mammospheres, suggesting that the effects of T3 on CSC biology are not related to EMT changes (114). By contrast, T3 inhibits the activation of NF-kB by TNFα in TRβ-expressing breast cancer cells (114). This reduced response could be involved in the downregulation of the CSC population by T3, since NF-kB is one of the pathways that govern stem cell expansion in response to cytokine release in the tumor microenvironment (120). Finally, TRβ has also been identified as an inhibitor of tumor lymphangiogenesis and sentinel lymph node invasion in breast cancer by a mechanism involving N-CoR-mediated silencing of pro-metastatic and lymphangiogenic genes, such as VEGF-C and VEGF-D (111), which are targets of NF-kB signaling pathway (121).

## Conclusions and perspectives

**Figure 1:**
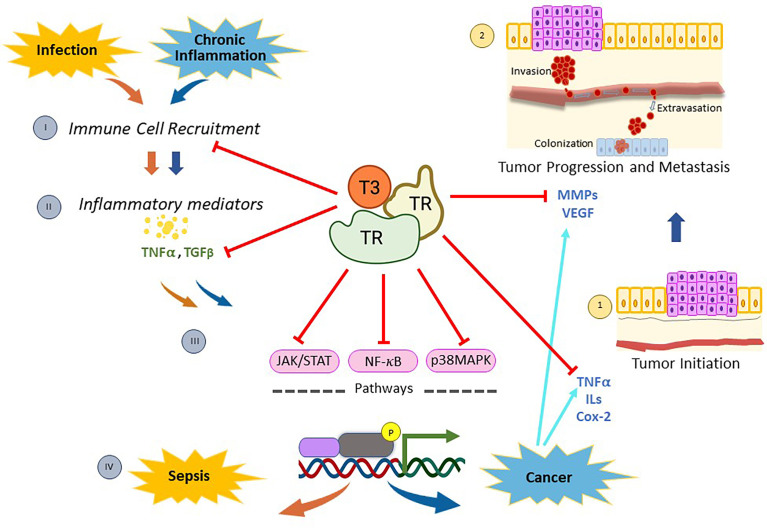
Mechanisms of action of thyroid nuclear receptors activated by T3 hormone on inflammation-related infection, and/or cancer. When pathogen infections occur, there is a recruitment of immune cells (I) that stimulate the synthesis and release of inflammatory mediators (II). Then, many of these molecules generate a genomic inflammatory response due to the activation of the signaling pathways mediated by NF-kB, p38MAPK, and/or JAK/STAT (III). If the infection persists, the host’s protective innate immunity is overactivated and a reaction called sepsis appears (IV). On the other hand, chronic inflammation is linked to diverse types of cancer through the production of inflammatory mediators (II), which activate the signaling pathways mentioned above (III) and induce the expression of several regulators of tumor initiation (1), progression and metastasis (2). Thyroid nuclear receptors (TRs) activated by T3 exert a protective role on both sepsis and cancer through several mechanisms. On one hand, TRs control the recruitment of immune cells and reduce the production of inflammatory mediators by down-regulating both NF-kB, p38MAPK and JAK/STAT pathways. In addition, TRs inhibit tumor initiation (1) by decreasing proliferation and inducing apoptosis through deregulation of expression of inflammatory mediators, such as TNFα and Cox-2. Moreover, TRs inhibit tumor progression and metastasis (2) by down-regulation of MMPs and/or VEGF. All these data demonstrate that T3 hormone through its binding to specific nuclear receptors counteracts the inflammation associated with cancer and/or infection, opening new lines of research and drug development to improve current therapeutic interventions in those patients.

In this review we have discussed the crosstalk between the immune system and THs, focusing on the relevance of activated nuclear TRs in the molecular processes involved in the inflammation associated with various infectious and/or pathological processes, such as cancer (**Figure 1**). In particular, we have emphasized the effects of TRs on molecular processes that are regulated by either NF-kB, p38MAPK and/or JAK/STAT signaling pathways, since they have been shown to play important roles in inflammation. On one hand, we summarize the important role of TRs as tumoral suppressors, counteracting different steps of the promotion and progression of a number of cancers by affecting several inflammatory mediators related to the pathways mentioned above. Moreover, we also provide evidence of the beneficial role of THs, through their binding to specific nuclear receptors, against the systemic inflammatory response of endotoxemia. To date, no specific drugs have been developed for the treatment of sepsis, nor has any biomarker been established that can definitively diagnose sepsis or predict its clinical course. In addition, no reliable tools for stratified management of patients with severe COVID-19 are available. On the other hand, there is a need for developing new strategies to bypass side effects associated with many treatments for cancer. The findings discussed in this review about the protective roles of TRs in models of these diseases open new lines of research and drug development to improve current therapeutic interventions with alternative strategies based on THs levels and/or TR expression in patients with inflammation-linked infections and/or diseases.

## Author contributions

All authors listed have made a substantial, direct, and intellectual contribution to the work and approved it for publication.

## Acknowledgments

This publication was supported by Fundación Universidad Alfonso X El Sabio.

## Conflict of interest

The authors declare that the research was conducted in the absence of any commercial or financial relationships that could be construed as a potential conflict of interest.

## Publisher’s note

All claims expressed in this article are solely those of the authors and do not necessarily represent those of their affiliated organizations, or those of the publisher, the editors and the reviewers. Any product that may be evaluated in this article, or claim that may be made by its manufacturer, is not guaranteed or endorsed by the publisher.
